# Fragment Localized Molecular Orbitals

**DOI:** 10.1021/acs.jctc.2c00359

**Published:** 2022-07-27

**Authors:** Tommaso Giovannini, Henrik Koch

**Affiliations:** † Scuola Normale Superiore, Piazza dei Cavalieri 7, 56126 Pisa, Italy; ‡ Department of Chemistry, Norwegian University of Science and Technology, 7491 Trondheim, Norway

## Abstract

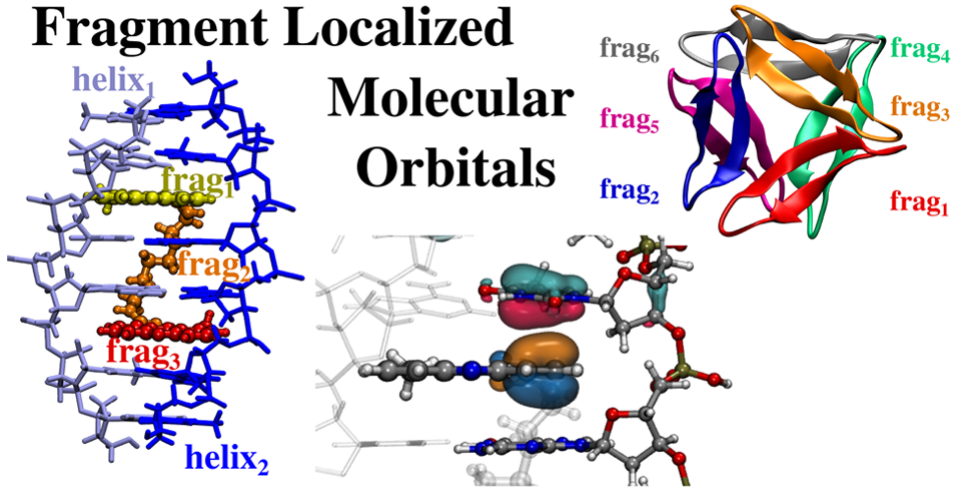

We introduce the concept of fragment localized molecular orbitals (FLMOs), which are Hartree–Fock molecular orbitals localized in specific fragments constituting a molecular system. In physical terms, we minimize the local electronic energies of the different fragments, at the cost of maximizing the repulsion between them. To showcase the approach, we rationalize the main interactions occurring in large biological systems in terms of interactions between the fragments of the system. In particular, we study an anticancer drug intercalated within DNA and retinal in anabaena sensory rhodopsin as prototypes of molecular systems embedded in biological matrixes. Finally, the FLMOs are exploited to rationalize the formation of two oligomers, prototypes of amyloid diseases, such as Parkinson and Alzheimer.

## 1 Introduction

Intermolecular interactions play a crucial role in many chemical and biological processes.^1^ For instance, they are the guiding force of different metabolic pathways and regulate the physicochemical properties of many materials and their interaction with light.^2−4^ A theoretical understanding of intermolecular interactions, together with the different energy contributions constituting them, is of particular importance, providing a solid base on which novel experimental setups/reactions can be designed. In the last decades, the size of the molecular systems which can be treated computationally has dramatically increased aided by developed methods with a favorable scaling with the system size.^5−8^ Such approaches has allowed for the description of more and more complex systems, without renouncing accuracy. In particular, they are typically based on chemical intuition which guides a hierarchical partitioning of the investigated system.^9−11^ Such approximations are justified by the assumption that the property of interest (energetics and/or spectroscopy) is usually local and carried by a specific part of the system, as in the case of local electronic excitations.^11−16^ To rationalize local properties, the standard procedure is to resort to localized molecular orbitals (LMOs) which usually act as a bridge between chemical intuition and theoretical chemistry.^17−21^ The success of LMOs is demonstrated by the plethora of applications in which they have been used by both theoretical and experimental groups.^15,22−25^

In this work, we introduce a novel class of LMOs, which are localized in specific fragments and/or portions of molecular systems. For this particular feature, we call them fragment localized molecular orbitals (FLMOs). The potential of FLMOs is evident: the local properties and/or energetics of different fragments interacting together can be calculated without resorting to any kind of approximation. The FLMOs are defined in the Hartree–Fock (HF) context. The idea behind our approach is rather simple: by reformulating the HF energy for a system constituted by several fragments, we minimize the local electronic energies of each of them, by keeping the total energy of the system unchanged. Therefore, such a minimization is carried out at the cost of maximizing the repulsion between the fragments and consequently providing the MOs mostly localized within the different fragments. Our approach is a top-down variational methodology, since the localization is performed by starting from the full canonical MOs. For the aforementioned reason, it differs from other approaches proposed in the literature to deal with fragment localized MOs.^26−31^ Note also that the term FLMO has been first proposed in ref (31), where fragment localized MOs are obtained in terms of the subsystems’ MOs previously determined, i.e., in a “fragment to molecule” fashion.

In this paper, we propose an application of FLMOs to the calculation of interaction energies of interesting molecular systems and, in particular, a decomposition of the interaction energy among the FLMOs belonging to different fragments is presented. Such a decomposition can provide a graphical and intuitive interpretation of the interactions among the interacting systems, which can guide both theoretical and experimental chemists in the in-silico design of novel processes. To showcase how FLMOs can be used in chemical and biological relevant applications, we study the fragment interaction energies of an anticancer drug, whose mechanism is evinced in its intercalation within DNA,^32^ in all trans-retinal embedded in a rhodopsin protein environment,^33^ as relevant biological applications. Finally, we investigate the molecular interactions within two oligomers, prototypes of the packaging involved in amyloid diseases, such as Parkinson or Alzheimer.^34−36^

## 2 Theoretical Method

The starting point of our localization procedure is the Hartree–Fock (HF) energy of a generic molecular system^37^
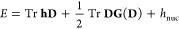
1where **h** and **G** are the usual one- and two-electron matrices, **D** is the density matrix, and *h*_nuc_ is the nuclear repulsion.^6^ If the system is partitioned in *N*_f_ fragments, the density matrix **D** can be decomposed as^21^
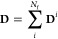
2where **D**^*i*^ is the density matrix of the *i*th fragment. The total energy defined in eq 1 can be rewritten as
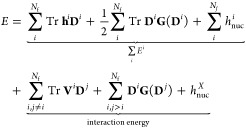
3where  is the nuclear repulsion between the *N*_f_ fragments, defined as . The **h**^*i*^ and **G**(**D**)^*i*^ matrices are defined as
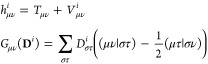
4where *T*_μν_ and  are the kinetic and electron–nuclear attraction matrix elements expressed in the full AO basis ({χ_μ_}), respectively. Note that in eq 3, the energy of the *i*th fragment and the interaction energy between all *N*_f_ fragments have been highlighted.

In order to localize the MOs in the predefined fragments, we can minimize the sum of the fragment energies (*E*^*i*^) as defined in eq 3 by rotating the occupied orbitals belonging to the different fragments, thus conserving the total energy of the system. This clearly corresponds to maximizing the interaction energy and therefore the repulsion between the fragments. In physical terms, this means that the minimization of the local electronic energies of the fragments can be carried out at the cost of maximizing the repulsion between them. From a computational point of view, we solve this problem by performing *N*_f_ – 1 different self-consistent field (SCF) steps. At the first step, the MOs of the first fragment are localized by maximizing the repulsion with all the other fragments. This step is performed in the MO space spanned by the occupied orbitals of all the fragments. Once the first fragment’s occupied MOs are localized, the process is repeated for all the other fragments, by reducing at each step the MO space in which the SCF procedure is performed. In fact, at the each cycle the MO space is reduced, because the occupied MOs localized at the previous step are removed. In this way, at the final *N*_f_ – 1 step, all the occupied MOs, which are expressed in the full AO basis, are still orthogonal and localized in their predefined fragments. For this reason, we propose to call them fragment localized molecular orbitals (FLMOs). It is worth remarking that after a fragment localization is performed, the following SCF cycles only rotate the occupied orbitals of the nonlocalized fragments, thus not affecting their total density. Note that, since the procedure involves *N*_f_ – 1 steps, the whole computational costs of the procedure is *N*_f_ · *t*_SCF_, where *t*_SCF_ is the timing associated with each SCF procedure. Note that the *t*_SCF_ associated with each SCF step can be significantly reduced by exploiting the procedure recently proposed by Koch and co-workers.^38^ The computational protocol is summarized in section S1 given in the Supporting Information, where we provide practical examples for covalently and noncovalently bonded systems.

One of the crucial steps in our procedure is the definition of the starting fragment MOs which are then localized. They are obtained by performing a partial Cholesky decomposition of the total density matrix at each localization step. Such a procedure has recently been detailed by us in ref (39), in which we also demonstrate that such a partitioning method guarantees the continuity of the potential energy surface (PES), differently from other localization techniques such as Boys.^18^ It is worth noting that the methodology here presented is completely general and any partitioning method can be used to obtain the starting MOs. However, the absence of any discontinuities in the PES must be carefully checked.^15^

Once the FLMOs are obtained, the corresponding fragment density matrices (FDM) can be constructed
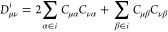
5where α and β labels run over the doubly and singly occupied MOs, respectively, both belonging to the *i*th fragment. For instance, in the case of a single covalent bond between two fragments is cut, we may assign one electron to each fragment sharing the chemical bond (see also section S1.2 in the Supporting Information). Both the FLMOs and FDMs are defined on the full AO basis set {χ_μ_}. This means that they may be characterized by tails in other fragments; however, such a property guarantees the orthogonality between them and the correct reproduction of the full HF results.

As stated above, the FLMOs which are obtained with our procedure may have different applications, ranging from reactivity to reduce the computational time associated with correlated methods. In this work, we decide to exploit them to the particular case of interaction energies. Once the FDMs have been computed, the interaction energy between the *i*th and *j*th fragments  can be defined as
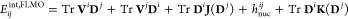
6
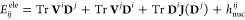
7
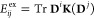
8where **J** and **K** are Coulomb and exchange matrices. In eqs 7 and 8, the different terms are grouped into the electrostatic  and the exchange  energy terms. The latter is always negative and therefore a stabilizing interaction. Note that the separation proposed in eqs 6–8 share the same formalism of other energy decomposition analysis.^40^ We can define the total interaction energy between the *i*th and *j*th fragments  as
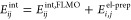
9
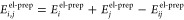
10where the electronic preparation energy  is the energy needed to deform the electron density of the *i*th fragment from the vacuo to the supramolecular structure, whereas  indicates that of the dimer *ij*. Further details about eqs 9 and 10 are given in section S1.5 in the Supporting Information. For a generic monomer or dimer *r*, the  is defined as the difference between the energy when *r* is embedded in the total system , and *r* energy in the gas-phase :
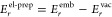
11

It is worth noting that for open-shell fragments, the vacuum calculations are computed at the unrestricted HF level. Moreover, the basis set superposition error (BSSE) can be easily reduced by using counterpoise correction, i.e., by calculating the vacuum energies (eq 11) in the full AO basis set.^41^

The total interaction energy between all fragments  can be defined by considering the energetic cost associated with the electronic preparations of the single monomers:
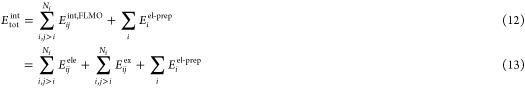
12where the electrostatic and exchange energy contributions are specified. The total interaction energy defined in eq 12 is by definition equal to the full HF case (see also section S1.5 in the Supporting Information). Finally, we note that eqs 5–8 can also be specified for two MOs belonging to two different fragments, thus allowing a further partitioning of the electrostatic and exchange contributions among the FLMOs of the two different fragments (see also section S1.1 in the Supporting Information). In this way, the interaction energy between two fragments can be rationalized in terms of the FLMOs belonging to the two fragments. In particular, we can decompose the interaction energy between two FLMOs in the electrostatic and exchange interactions, thus providing a physicochemical insight into the interactions.

## 3 Numerical Applications

To demonstrate the robustness and the potential of the method, we select two test applications: two molecular systems embedded in a biological matrix (DNA or protein)^32,33^ and two different prototypes for the amyloid diseases.^34^ For all systems, we first perform the FLMO localization, and then we decompose the interaction energies among the different terms as defined in eq 6. The FLMO decomposition is implemented in a development version of the electronic structure code .^42^ Due to the size of the systems, the dispersion interaction is treated by Grimme’s D3 method, which is an effective parameter-dependent approach,^43^ thus it does not depend on the computed FLMOs. All calculations are performed on 28 processors Intel Xeon Gold 5120 CPU at 2.20 GHz.

### 3.1 Molecular Systems Embedded in Biological Matrixes

**Figure 1:**
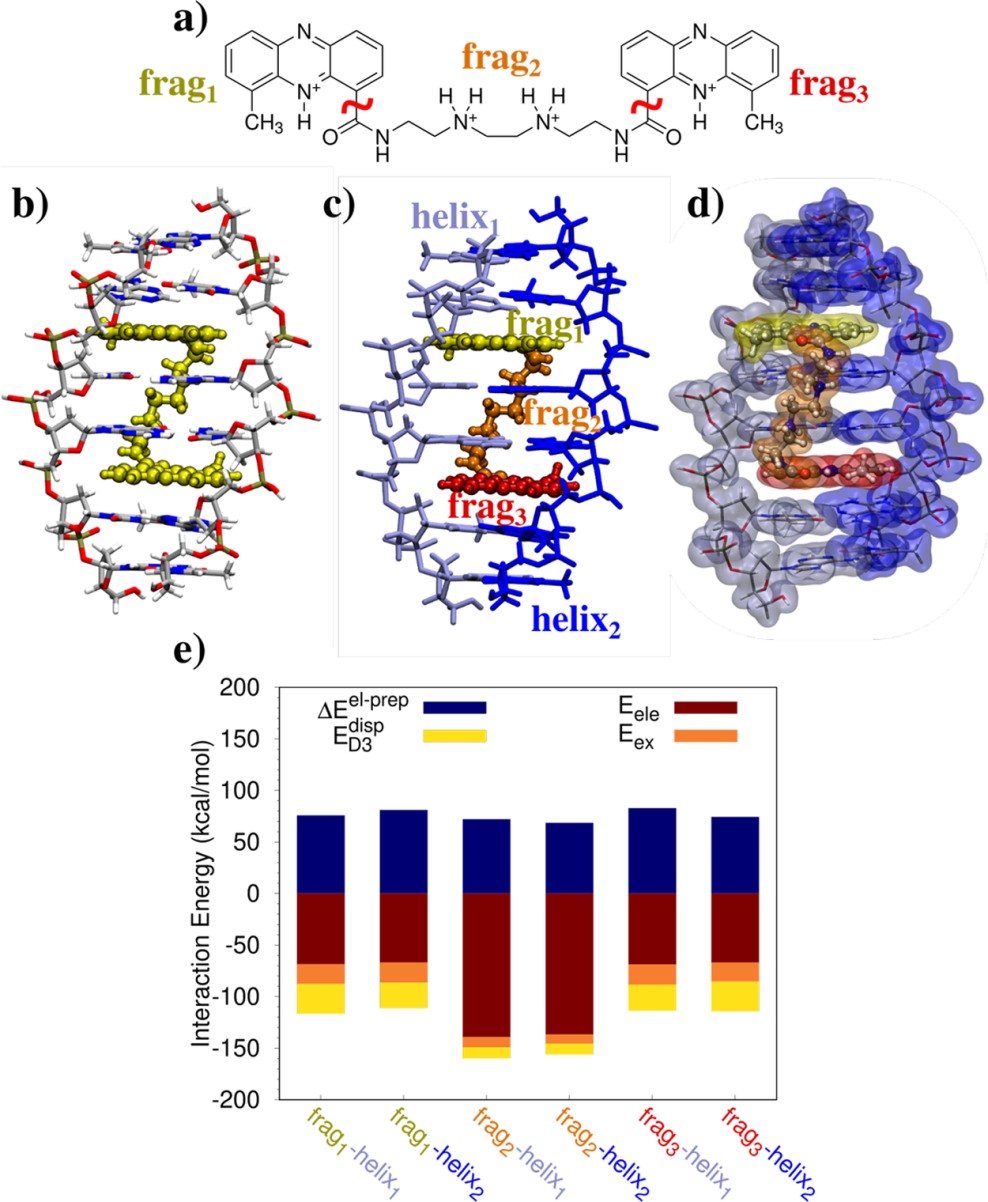
(a) Molecular structure of the studied drug (XR5944). (b) XR5944 intercalated within DNA (PDB 1X95), (c) the exploited fragmentation, and (d) the fragment densities obtained by using the FLMOs procedure. (e) FLMOs interaction energy decomposition analysis (kcal/mol).

The first studied system is XR5944, an anticancer drug which is constituted of an aliphatic chain connecting two aromatic groups (see Figure 1a).^32,44−46^ Similar to many other anticancer drugs,^47,48^ the drug mechanism is based on the intercalation between two DNA basis pairs (see Figure 1b; PDB 1X95), in this way avoiding its duplication and the diffusion of the cancer. The physicochemical understanding of the main forces binding the molecular drug to the DNA can be of great relevance since it can guide the design of novel drugs with the same mechanism. To this end, we divide the drug in three open shell fragments: the two aromatic rings and the aliphatic chain (see Figure 1a,c). The two DNA helices are instead treated as two different fragments (helix_1_ and helix_2_). Finally, we compute the FLMOs and the interaction energy components at the HF/6-311G* level (total number of AOs, *N*_AO_ = 5756, average *t*_SCF_ = 1484 s). The resulting fragment densities, localized in their spatial regions, are reported in Figure 1d. The interaction energy components between the three drug fragments and the two helices are graphiscally depicted in Figure 1e (see Table S1 in the Supporting Information for raw data).

In all cases, the electrostatic interaction *E*_ele_ is negative, thus stabilizing the interaction of the drug with the DNA. For frag_1_ and frag_3_, i.e., the two aromatic rings, *E*_ele_ is always opposite to the purely repulsive contribution Δ*E*^el-prep^, which, when summed with *E*_ex_ represents the exchange-repulsion term (*E*_ex-rep_), i.e., the Pauli repulsion.^49^ For all the energetic contributions, the symmetry of the system is reflected in a symmetry of the energy terms, with frag_1_ and frag_3_ interacting most favorably with helix_1_ and helix_2_, respectively. Also, frag_2_ is interacting in a symmetrical way with the two helices, with the total interaction energies differing by just 0.2 kcal/mol. Interestingly, the predominant contribution is the electrostatics, which is more than twice the corresponding energy terms computed for both frag_1_ and frag_3_. This makes the interaction of frag_2_ with the DNA the leading interaction allowing for the intercalation, or at least the stabilization, of the drug within the DNA. This is the theoretical reason for which many anticancer drugs are designed with a similar lateral chain, which acts as an anchor hydrogen-bonded with the DNA, stabilizing the complex.

**Figure 2:**
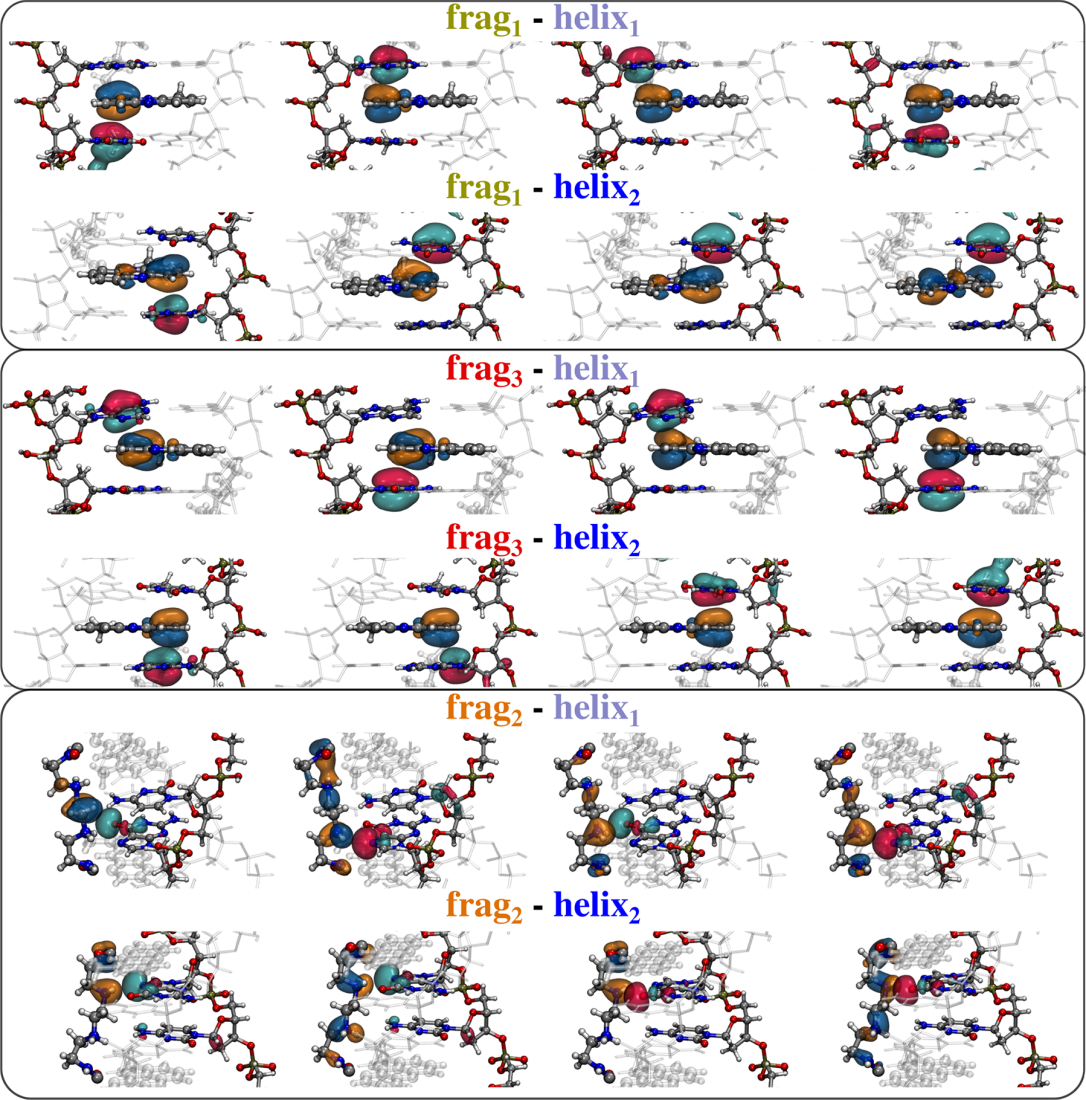
Graphical representation of the most important FLMOs interactions for all the studied fragment couples (top, frag_1_-helix_1,2_; middle, frag_3_-helix_1,2_; bottom, top: frag_2_-helix_1,2_).

As detailed above, our method also allows for a qualitative and quantitative investigation of the interaction by decomposing both *E*_ele_ and *E*_ex_ in terms of the FLMOs belonging to different fragments. In Figure 2, we report the four most relevant FLMOs contributing to *E*_ex_ for each studied pair. Note that the computed FLMOs are characterized by a localization spread comparable to what has been reported by the authors in a recent paper.^39^ As expected, the top and middle panels show that the interaction between the two aromatic rings (frag_1_ and frag_3_) and both DNA helices is dominated by π–π stacking. Also, the aforementioned geometrical symmetry is displayed by the FLMOs that dominate the interactions, which are almost identical for frag_1_-helix_1_ and frag_3_-helix_2_. A different situation is depicted for frag_2_ for which a direct hydrogen-bonding is clearly evident in all selected FLMOs. Such a qualitative analysis provides an intuitive theoretical explanation for the almost 2-fold value of the total interaction energy obtained for the lateral chain as compared to the two aromatic rings of the drug.

**Figure 3:**
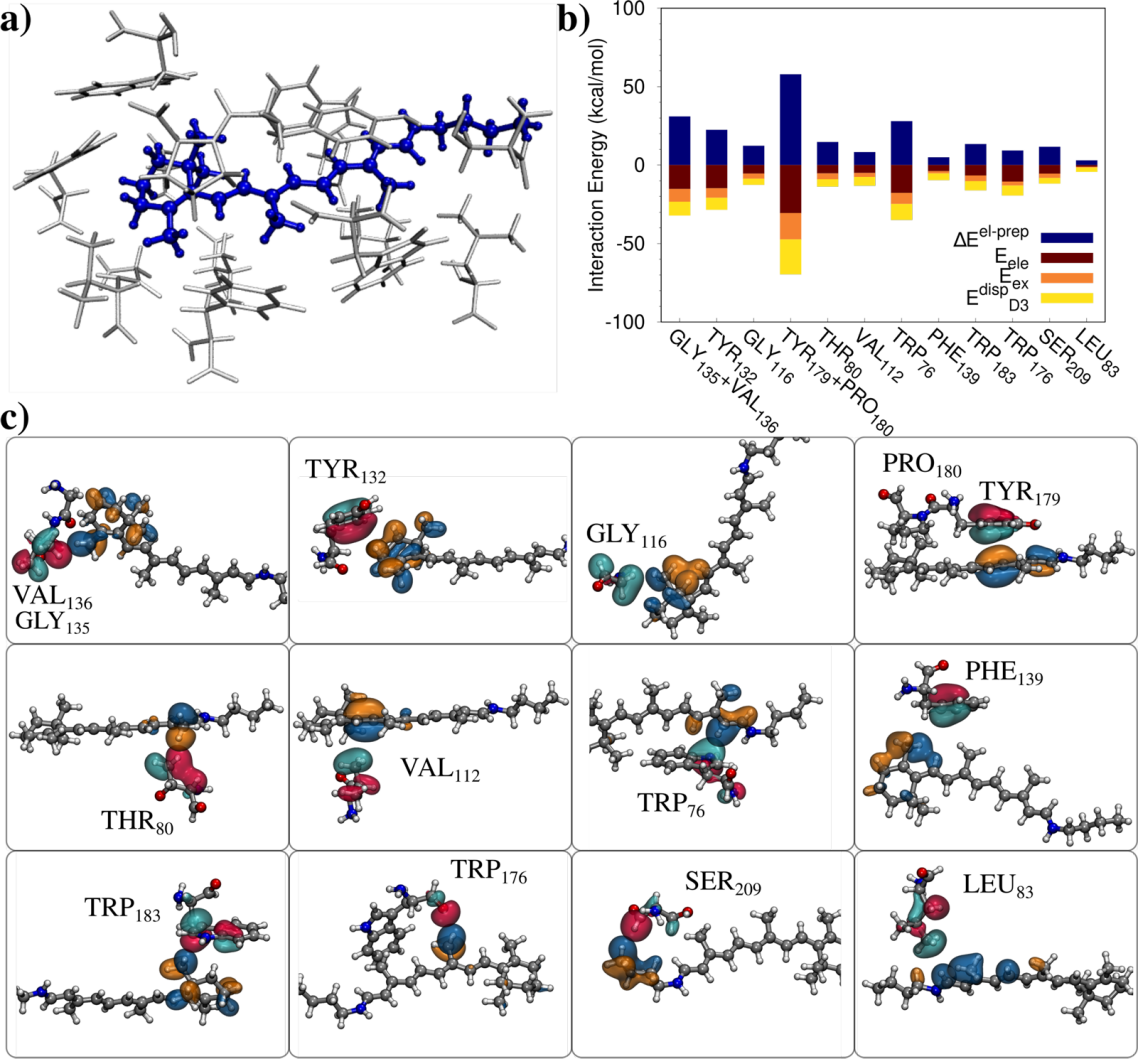
(a) Molecular structure of all-trans retinal chromophore embedded in the closest 14 amino acids of the anabaena sensory rhodopsin.^33^ (b) FLMOs interaction energy decomposition analysis (kcal/mol). (c) Graphical representation of the most important FLMOs interactions between retinal and the 14 selected amino acids.

The second studied system is the all-trans retinal chromophore embedded in anabaena sensory rhodopsin (ASR), a light sensor, as reported in ref (33), by Olivucci and co-workers. The system is particularly interesting because it is the chromophore responsible for the fluorescence activity of the considered rhodopsin, thus permitting its potential use in the field of optogenetics via a modification of some peptides.^33^ In this work, we showcase our novel approach by selecting retinal and the closest 14 amino acids in its wild type form, which are described at the HF/cc-pVDZ level (total *N*_AO_ = 2993, average *t*_SCF_ = 491 s; see Figure 3a). Differently from the previous case, we consider closed shell fragments, i.e., if two amino acids are covalently bonded, they are treated as a single fragment, resulting in a total of 12 fragments. In this way, we are also demonstrating the flexibility of our approach. After localizing the FLMOs, we calculate and decompose the interaction energy between retinal and the different fragments (see Figure 3 and Table S2 in the Supporting Information for raw data). Also in this case, the electrostatic interaction is attractive for all the considered fragments; however, the magnitude of the interaction is much lower as compared to that obtained in case of XR5944 and the DNA. In order to further investigate such a difference, the most relevant FLMOs interactions for each retinal–amino acid pair are graphically depicted in Figure 3c. Clearly, in any pair, no hydrogen bondings are present. However, a π–π interaction is only depicted in the case of TYR_179_ + PRO_180_ fragment, in which the aromatic ring of TYR is stacked together with the double bonds of retinal. This is the reason why for such a fragment, the interaction energy components are larger than all the other fragments. Interestingly for almost all fragments, the exchange-repulsion term exceeds the electrostatics; however, the interaction energy is always attractive (except for THS_80_). Therefore, in this system, dispersive contributions play a leading role. Finally, we notice that a large interaction energy is reported also for TRP_176_, for which *E*^ele^ exceeds *E*_ex-rep_ (in absolute value). By inspecting Figure 3c, this is probably due to the favorable electrostatic interaction between the peptide C=O group and the retinal.

### 3.2 Prototypes of Amyloid Diseases

**Figure 4:**
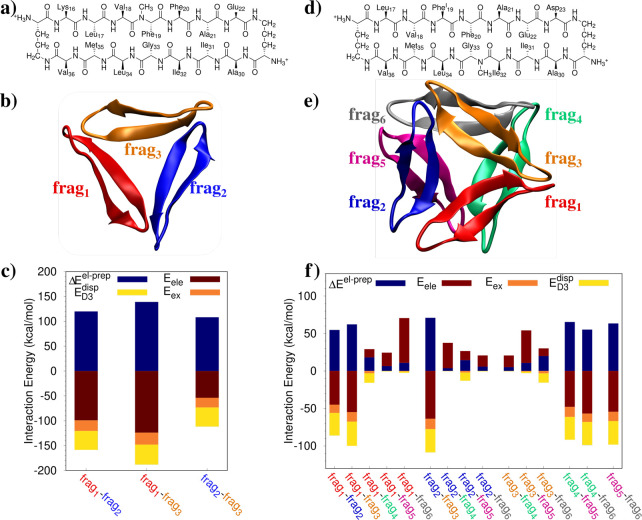
Molecular structures of the selected amyloid peptide prototypes (a–d) and their three-dimensional arrangements (b–e). FLMOs interaction energy decomposition analysis (parts c–f, kcal/mol).

As a final test case, we study two different prototypes of the amyloid diseases, such as Alzheimer, Parkinson, and type 2 diabetes.^34^ In particular such diseases are historically related to the formation of plaques and fibrils caused by β-amyloid peptide Aβ and recently to the formation of small aggregates, called oligomers.^34^ Special efforts on the last direction have been paid by the Nowick group^34^ that has designed different β-sheets to mimic the β-hairpins typical of amyloid peptides. In this work, we select a trimer assembly (see Figure 4a–c, PDB 5W4H), which is seen as a unprecedented mode of self-assembly,^50^ and an hexamer oligomer (see Figure 4d–f, PDB 5V65), which is one of the possible higher-order assemblies of the trimer unit.^51^ We aim at calculating the main energy components defining the interaction of the considered systems, in order to provide a first theoretical glimpse of the formation of such oligomers. The study of the interactions between the macrocycles may be of particular importance to understand the leading force that bring them to agglomerate, a possible cause of the aforementioned diseases.

To this end, each oligomer is treated as a separate fragment, and the interaction between the different groups is calculated after the FLMOs are obtained by exploiting our presented procedure. In both the trimer and hexamer assemblies, the total system is described at the HF/6-311G* level (trimer: *N*_AO_ = 8079 (average *t*_SCF_ = 7158 s); hexamer: *N*_AO_ = 16224, average *t*_SCF_ = 21086 s). We first focus our attention on the trimer case, in which the three peptides are constituted by two Aβ_16–22_ and Aβ_30–36_ β-sheets, which are bonded by two ^δ^Orn units. Notice that an N-methyl group is placed on Phe_19_.^50^ The trimer packaging is depicted in Figure 4b, and the decomposition of the interaction energy between the three monomers is graphically depicted in Figure 4c (see Table S3 in the Supporting Information for raw data). The electrostatic interaction is always attractive and for frag_1_-frag_2_ cancels out with the exchange-repulsion energy term. In all three cases, the total interaction energy is attractive because of the presence of the dispersion energy term, whose contribution ranges from 32% to 72% with respect to electrostatics. Therefore, intermolecular hydrogen bonds between the monomers play a crucial role; however, its covalent contribution, which is contained in the exchange-repulsion term, makes dispersion interactions crucial for the packaging of the system. Notice that our analysis thus suggests that the packaging is energetically favored in addition to hydrophobic forces^52^ that are surely in play for such a system.

Finally, the hexamer assembly obtained by the packaging of the peptide depicted in Figure 4d is considered.^51^ Hexamers, and generally high-order oligomers, are formed by trimers which assemble together to create hydrophobic pockets. In particular, the peptide monomer is constituted by two Aβ_17–23_ and Aβ_30–36_ β-sheets which, again, are linked by two ^δ^Orn units. In this case, Phe_19_ is substituted with a iodine atom. Similarly to the previous case, we study the interaction between the six fragments by means of the different energy components (see Figure 4f and Table S4 in the Supporting Information for raw data). First, we notice that the involved energies are generally lower than the trimer case. This is the result of the larger distance between the fragments in the hexamer arrangement. Figure 4f clearly shows that the electrostatic interaction between the fragments is attractive for the considered fragments being part of the same trimer (frag_1-3_ and frag_4-5_), whereas the opposite holds for the fragments belonging to the different trimers. Indeed, the total interaction between the two trimers is repulsive because no hydrogen bonds can energetically stabilize the structure, whereas the interaction between the peptides constituting the trimer is attractive. Therefore, our analysis shows that the first formation of the trimers is also energetic-guided; however, higher-order oligomers only appear because of hydrophobic forces.

## 4 Conclusions

In summary, we have introduced the concept of FLMOs, which are the result of a variational optimization of the energetics of different fragments defining a molecular system, either covalently bonded or composed by different moieties. The FLMOs can be exploited for a plethora of applications, ranging from reactive chemistry and spectroscopy, as for instance, by reducing the computational cost associated with highly correlated methods. In this paper, we showcase FLMOs potential by proposing a simple energy decomposition analysis based on FLMOs, which is applied to systems of significant chemical and biological interest. In particular, we have demonstrated that many anticancer drugs intercalate within DNA thanks to the lateral chain which acts as an anchor through hydrogen bonding interactions. Then, we have studied all-trans retinal embedded in a rhodopsin protein, showing that π–π interactions play the most relevant role in the interaction between the two systems and likely determine the molecular response of the chromophore. Finally, we have investigated two oligomers, prototypes of amyloid diseases. In particular, we have shown that the aggregation of the trimer form, which is the basis for higher-order oligomers, is energetically favored. On the contrary, the coupling between different trimers takes place because of hydrophobic forces. Within our theoretical approach, we have showed how FLMOs can be used in practical applications and can aid further design and deeper understanding of complex phenomena, such as the formations of plaques in brain diseases.

## Supporting Information Available

Computational protocol, FLMOs energy decomposition, and raw data for Figures 1–4 (PDF)
