# Supplementary material for: Fragment Localized Molecular Orbitals
Source: J Chem Theory Comput. 2022 Jul 27;18(8):4806–13. doi: 10.1021/acs.jctc.2c00359 (PMC9367013; doi:10.1021/acs.jctc.2c00359)
Supplement: Supplementary file 1 — ct2c00359_si_001.pdf [file ct2c00359_si_001.pdf]

Supporting Information: Fragment Localized Molecular Orbitals

Tommaso Giovannini*,† and Henrik Koch*,†,‡

†*Scuola Normale Superiore, Piazza dei Cavalieri 7, 56126 Pisa, Italy.*

‡*Department of Chemistry, Norwegian University of Science and Technology, 7491 Trondheim, Norway*

E-mail: tommaso.giovannini@sns.it; henrik.koch@sns.it

S1 Computational Protocol

S1.1 Non-Covalently Bonded Fragments

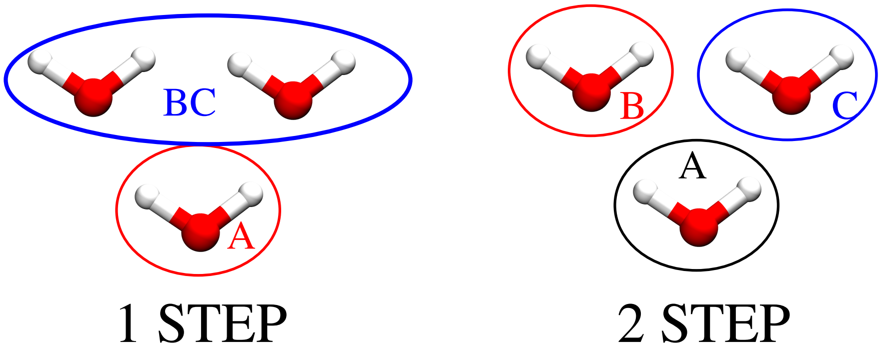

Figure S1: Three fragment scheme

Let us consider the three fragment scheme in Fig. S1. Let us divide the system in the parts: A and $B + C$. Then, the A density can be localized by following MLHF-AB scheme. In particular, the sum of the energies of the two parts reads:

$$E^A + E^{BC} = \text{Tr } \mathbf{h}^A\mathbf{D}^A + \text{Tr } \mathbf{h}^{BC}\mathbf{D}^{BC} + \frac{1}{2}\text{Tr } \mathbf{D}^A\mathbf{G}(\mathbf{D}^A) + \frac{1}{2}\text{Tr } \mathbf{D}^{BC}\mathbf{G}(\mathbf{D}^{BC}) \quad \text{(S1)}$$

where, $BC = B + C$, and the same holds for the corresponding density matrices. By using $\mathbf{D} = \mathbf{D}^A + \mathbf{D}^{BC}$ then, eq. S1 becomes:

$$E^A + E^{BC} = \text{Tr } (\mathbf{V}^A - \mathbf{V}^{BC})\mathbf{D}^A + \text{Tr } \mathbf{D}^A\mathbf{G}(\mathbf{D}^A) - \text{Tr } \mathbf{D}^A\mathbf{G}(\mathbf{D}) + \text{Tr } \mathbf{h}^{BC}\mathbf{D} + \frac{1}{2}\text{Tr } \mathbf{D}\mathbf{G}(\mathbf{D}) \quad \text{(S2)}$$

$$\mathbf{F}^A = \mathbf{V}^A - \mathbf{V}^{BC} + 2\mathbf{G}(\mathbf{D}^A) - 2\mathbf{G}(\mathbf{D}) \quad \text{(S3)}$$

which is exactly the same formulation proposed for MLHF-AB. After minimizing eq. S3,

we obtain two densities for A and BC systems which are maximally localized. However, B and C are not maximally localized in their specific spatial regions. To this end, we can now reformulate the problem as a true three fragment scheme, in which, however, $\mathbf{D}^A$ is kept frozen, and its orbitals do no enter in the occupied occupied rotations, which will be performed in the BC space only. However, the effect of $\mathbf{D}^A$ is taken into account by considering Eq. S4, which can be recasted in the following way:

$$
\begin{aligned}
E^A + E^B + E^C[\mathbf{D}^B] = &\ \mathrm{Tr}\ (\mathbf{V}^B - \mathbf{V}^C)\mathbf{D}^B + \mathrm{Tr}\ \mathbf{D}^B\mathbf{G}(\mathbf{D}^B) - \mathrm{Tr}\ \mathbf{D}^B\mathbf{G}(\mathbf{D}) + \mathrm{Tr}\ \mathbf{D}^B\mathbf{G}(\mathbf{D}^A) \\
&+ \underbrace{\mathrm{Tr}\ (\mathbf{V}^A - \mathbf{V}^C)\mathbf{D}^A + \mathrm{Tr}\ \mathbf{D}^A\mathbf{G}(\mathbf{D}^A) - \mathrm{Tr}\ \mathbf{D}^A\mathbf{G}(\mathbf{D})}_{\text{constant}} \\
&+ \underbrace{\mathrm{Tr}\ \mathbf{h}^C\mathbf{D} + \frac{1}{2}\mathrm{Tr}\ \mathbf{D}\mathbf{G}(\mathbf{D})}_{\text{constant}} \qquad \text{(S4)}
\end{aligned}
$$

from which the Fock matrix can be constructed:

$$
\mathbf{F}^B = (\mathbf{V}^B - \mathbf{V}^C) + 2\mathbf{G}(\mathbf{D}^B) - 2\mathbf{G}(\mathbf{D}) + 2\mathbf{G}(\mathbf{D}^A) \qquad \text{(S5)}
$$

S1.2 Covalently Bonded Fragments

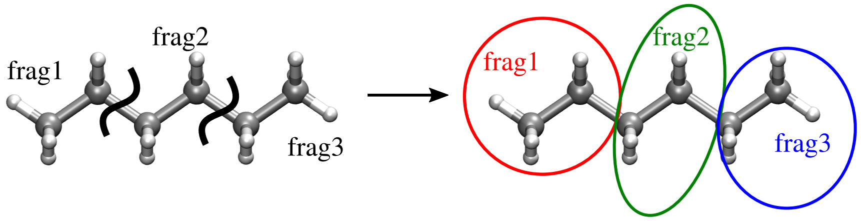

Figure S2: Example of electron assignment in Hexane as partitioned in three fragments.

We now consider the case in which the system is a supramolecule in which the different fragments are covalently bonded, via a single covalent bond. As explained in the main text, our procedure is carried out for closed shell fragments. Therefore, the user needs to define

to which fragment the bonding electrons have to be assigned. As an example, we show how such a partitioning can be done for the Hexane molecule in Fig. S2. In this case, we assign the bonding electrons between frag1 and frag2 to frag1, and those between frag2 and frag3 to frag2. Notice that the procedure is completely independent of such an assignment (vide infra). Once such a decision is taken, the computational protocol exactly follows what has already been explained in case of the water trimer. At the end of the procedure, there will be three fragment densities characterized by the pre-defined number of electrons. More importantly, for the fragments to which the bonding electrons were initially assigned, we will obtain some FLMOs resembling the bonding MOs. We provide a practical example for an ethane moiety described at the HF/aug-cc-pVDZ level (see Fig. S3).

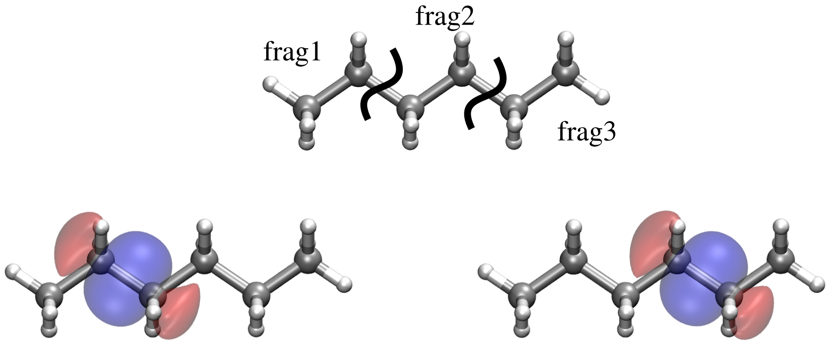

Figure S3: Hexane Molecule and bonding FLMO.

Once the bonding FLMOs are identified (such a step is very easy, since they are the highest in energy among the FLMOs of the considered fragment), the fragment densities of the two fragments covalently bonded can be updated by exploiting Eq. 5 in the main text (i.e. one electron is removed to one fragment density, and added to the other). Therefore, the procedure is independent of the initial electron assignment choice.

S1.3 N-fragment case

In general, at each cycle we are working in a reduced MO space, in which the occupied MOs of the previously optimized density are removed. Eq. S4 can be reformulated in case of N

densities, and for which we want to optimized the i-th density:

$$\sum_{j}^{N} E^j[\mathbf{D}^i] = \text{Tr } (\mathbf{V}^i - \mathbf{V}^{i+1\cdots N})\mathbf{D}^i + \text{Tr } \mathbf{D}^i\mathbf{G}(\mathbf{D}^i) - \text{Tr } \mathbf{D}^i\mathbf{G}(\mathbf{D}) + \sum_{k<i}^{N} \text{Tr } \mathbf{D}^i\mathbf{G}(\mathbf{D}^k)$$
$$+ \underbrace{\sum_{k<i} \left[\text{Tr } (\mathbf{V}^k - \mathbf{V}^{i+1\cdots N})\mathbf{D}^k + \text{Tr } \mathbf{D}^k\mathbf{G}(\mathbf{D}^k) - \text{Tr } \mathbf{D}^k\mathbf{G}(\mathbf{D}) + \sum_{l<k} \text{Tr } \mathbf{D}^k\mathbf{G}(\mathbf{D}^l) \right]}_{\text{constant}}$$
$$+ \underbrace{\text{Tr } \mathbf{h}^{i+1\cdots N}\mathbf{D} + \frac{1}{2}\text{Tr } \mathbf{D}\mathbf{G}(\mathbf{D})}_{\text{constant}} \tag{S6}$$

from which the Fock matrix can be constructed:

$$\mathbf{F}^i = (\mathbf{V}^i - \mathbf{V}^{i+1\cdots N}) + 2\mathbf{G}(\mathbf{D}^i) - 2\mathbf{G}(\mathbf{D}) + 2\sum_{k<i}^{N} \mathbf{G}(\mathbf{D}^k) \tag{S7}$$

To summarize, the procedure is characterized by the following points:

1. Construction of the $\mathbf{G}(\mathbf{D})$ and all the terms Tr $\mathbf{h}^{j\cdots N}\mathbf{D} = \text{Tr } (\mathbf{T} + \sum_{k\geq j}^{N} \mathbf{V}^k)\mathbf{D}$. In this way, we have at once all the constant terms due to the total density $\mathbf{D}$

2. Construction of one electron matrices at the i-th step.

3. From the previous cycle Fock matrix construct all the constant terms due to the $i-1$-th optimized density matrix.

4. Run the occupied-occupied optimization.

5. Readjust the Fragment Density according to Eq. 5 in the main text.

S1.4 Interaction Energy Decomposition among the FLMOs

To generalize the energy decomposition analysis among the Fragment-Localized Molecular Orbitals (FLMOs), we consider two orbitals α and β localized in fragment A and fragment

B, respectively. We assume that the two orbitals are doubly occupied; thus, two density matrices can be computed as:

$$D_{\mu\nu}^{\alpha} = 2C_{\mu\alpha}C_{\nu\alpha} \tag{S8}$$

$$D_{\mu\nu}^{\beta} = 2C_{\mu\beta}C_{\nu\beta} \tag{S9}$$

By following the derivation commented in the main text, the interaction between the two densities can therefore be written as:

$$E_{\alpha\beta}^{int,FLMO} = \frac{\operatorname{Tr}\mathbf{V}^{\alpha}\mathbf{D}^{\beta}}{n_o^A} + \frac{\operatorname{Tr}\mathbf{V}^{\beta}\mathbf{D}^{\alpha}}{n_o^B} + \operatorname{Tr}\mathbf{D}^{\alpha}\mathbf{J}(\mathbf{D}^{\beta}) + \frac{h_{nuc}^{\alpha\beta}}{n_o^A + n_o^B} - \operatorname{Tr}\mathbf{D}^{\alpha}\mathbf{K}(\mathbf{D}^{\beta}) \tag{S10}$$

$$E_{\alpha\beta}^{ele} = \frac{\operatorname{Tr}\mathbf{V}^{\alpha}\mathbf{D}^{\beta}}{n_o^A} + \frac{\operatorname{Tr}\mathbf{V}^{\beta}\mathbf{D}^{\alpha}}{n_o^B} + \operatorname{Tr}\mathbf{D}^{\alpha}\mathbf{J}(\mathbf{D}^{\beta}) + \frac{h_{nuc}^{\alpha\beta}}{n_o^A + n_o^B} \tag{S11}$$

$$E_{\alpha\beta}^{ex} = -\operatorname{Tr}\mathbf{D}^{\alpha}\mathbf{K}(\mathbf{D}^{\beta}) \tag{S12}$$

where, the factors n_o^A and n_o^B corresponds to the number of occupied MOs of fragments A and B, respectively. Such factors allows for the correct limit when all α and β occupied MOs of the two fragments are summed.

S1.5 Interaction Energies

Dimer The interaction energy for a generic dimer AB, composed of two monomers A and B, is defined as:

$$E_{AB}^{int} = E_{AB} - E_A^{vac} - E_B^{vac} \tag{S13}$$

where E_{AB} is the dimer energy, whereas E_A^{vac} and E_B^{vac} are the monomers energy in gas-phase (which can in principle be computed by including counterpoise correction). Note that, for

covalently bonded fragments, E_X^{vac} is calculated at the Unrestricted HF level. Equivalently, Eq. S13 can be recasted in terms of FLMO related quantities (see Eqs. 3,7 in the main text) as:

$$E_{AB}^{int} = E_{AB}^{int,FLMO} + E_A^{emb} + E_B^{emb} - E_A^{vac} - E_B^{vac} \tag{S14}$$

$$= E_{AB}^{int,FLMO} + (E_A^{emb} - E_A^{vac}) + (E_B^{emb} - E_B^{vac}) \tag{S15}$$

$$= E_{AB}^{int,FLMO} + E_A^{el-prep} + E_B^{el-prep} \tag{S16}$$

where E_{AB} has been rewritten in terms of A, B energies for the monomers embedded in the dimer ($E_A^{emb}+E_B^{emb}$), and their interaction energy calculated as reported in Eq. 6 in the main text. In Eq. S16, the electronic-preparation energies of the two monomers are introduced. Such an energetic term takes into account the cost to bring the two monomers from the gas-phase to the final electronic state of the dimer.

Trimer The interaction energy for a generic trimer ABC, composed of three monomers A, B and C, is defined as:

$$E_{ABC}^{int} = E_{ABC} - E_A^{vac} - E_B^{vac} - E_C^{vac} \tag{S17}$$

By using the same procedure exposed above (which is also valid for a general N fragment case), we obtain:

$$\begin{aligned} E_{ABC}^{int} &= E_{ABC}^{int,FLMO} + E_A^{emb} + E_B^{emb} + E_C^{emb} - E_A^{vac} - E_B^{vac} - E_C^{vac} \\ &= E_{ABC}^{int,FLMO} + E_A^{el-prep} + E_B^{el-prep} + E_C^{el-prep} \end{aligned} \tag{S18}$$

We can now introduce the three-body energy term (ΔE^{3B}), which reads:

$$\Delta E^{3B} = E^{ABC} - E_A^{vac} - E_B^{vac} - E_C^{vac} - E_{AB}^{int,vac} - E_{BC}^{int,vac} - E_{AC}^{int,vac} \tag{S19}$$

Therefore, Eq. S17 can be rewritten as:

$$E_{ABC}^{int} = E_{AB}^{int,vac} + E_{BC}^{int,vac} + E_{AC}^{int,vac} + \Delta E^{3B} \tag{S20}$$

By then considering that $E_{ABC}^{int,FLMO}$ is additive in the interaction energies between the dimers (see main text), we can compare Eqs. S18 and S20, which yields:

$$E_{AB}^{int,FLMO} + E_{BC}^{int,FLMO} + E_{AC}^{int,FLMO} + E_A^{el-prep} + E_B^{el-prep} + E_C^{el-prep} = E_{AB}^{int,vac} + E_{BC}^{int,vac} + E_{AC}^{int,vac} + \Delta E^{3B} \tag{S21}$$

and therefore, an expression for the three-body energy term (ΔE^{3B}) can be obtained:

$$\Delta E^{3B} = E_A^{el-prep} + E_B^{el-prep} + E_C^{el-prep} + E_{AB}^{int,prep} + E_{BC}^{int,prep} + E_{AC}^{int,prep} \tag{S22}$$

$$E_{XY}^{int,prep} = E_{XY}^{int,FLMO} - E_{XY}^{int,vac} \tag{S23}$$

We now move to calculate the interaction energy between two subsystems X and Y, which is defined as:

$$E_{XY}^{int} = E_{XY}^{vac} - E_X^{vac} - E_Y^{vac} \tag{S24}$$

Equivalently, it can be computed by using FLMO-based quantities as:

$$E_{XY}^{int} = E_{XY}^{int,FLMO} + E_{X,Y}^{el-prep} \tag{S25}$$

$$E_{X,Y}^{el-prep} = E_{X}^{el-prep} + E_{Y}^{el-prep} - E_{XY}^{el-prep} \tag{S26}$$

$$E_{XY}^{el-prep} = E_{XY}^{emb} - E_{XY}^{vac} \tag{S27}$$

where $E_{XY}^{el-prep}$ is the electronic preparation energy of the dimer. By using S25, the interaction energy between two dimers can be decomposed in terms of FLMO-based quantities.

It is worth noting that in case the three fragments are covalently bonded, e.g. the hexane molecule depicted in Fig. S2, the gas-phase energies of the different fragments are computed at the UHF level, by specifying the correct multiplicity. On the other hand, E_{X}^{emb} are calculated by following the procedure described in Sec. S1.2 and by exploiting Eq. 5 in the main text.

S2 Results

Table S1: Interaction energy decomposition analysis (kcal/mol) between the three fragments constituting XR5944 and the two helix of the DNA.

Fragments	E_{ele}	E_{ex}	$\Delta E^{el-prep}$	E_{D3}^{disp}	E_{int}
$frag_1$-$helix_1$	-68.6	-19.2	75.7	-28.9	-41.0
$frag_1$-$helix_2$	-66.9	-19.7	81.0	-24.5	-30.1
$frag_2$-$helix_1$	-139.4	-9.9	72.0	-10.6	-87.8
$frag_2$-$helix_2$	-136.8	-9.1	68.5	-10.3	-87.6
$frag_3$-$helix_1$	-68.8	-19.8	82.7	-25.0	-30.9
$frag_3$-$helix_2$	-66.9	-18.6	74.2	-28.7	-39.9

Table S2: Interaction energy decomposition analysis (kcal/mol) between all-trans retinal and the closest 14 aminoacids of the Anabaena Sensory Rhodopsin.

Fragments	E_{ele}	E_{ex}	$\Delta E^{el-prep}$	E_{D3}^{disp}	E_{int}
GLY_{135}+VAL_{136}	-15.2	-8.1	31.0	-8.7	-1.1
TYR_{132}	-14.8	-6.1	22.4	-7.6	-6.1
GLY_{116}	-5.4	-3.3	12.3	-3.8	-0.3
TYR_{179}+PRO_{180}	-30.6	-16.8	57.8	-22.2	-11.8
THR_{80}	-5.3	-3.7	14.7	-4.7	0.9
VAL_{112}	-5.0	-2.5	8.3	-5.6	-4.9
TRP_{76}	-17.8	-7.0	28.0	-10.2	-7.0
PHE_{139}	-3.9	-1.4	4.9	-4.3	-4.7
TRP_{183}	-6.7	-3.6	13.4	-5.9	-2.7
TRP_{176}	-10.5	-2.6	9.2	-6.3	-10.1
SER_{209}	-5.4	-2.8	11.6	-3.6	-0.2
LEU_{83}	-1.0	-0.7	3.0	-2.6	-1.3

Table S3: Interaction energy decomposition analysis (kcal/mol) between the three peptides constituting the trimer assembly, prototype of amyloid peptide (see fig. 4 a,b,c).

Fragments	E_{ele}	E_{ex}	$\Delta E^{el-prep}$	E_{D3}^{disp}	E_{int}
$frag_1$-$frag_2$	-99.2	-21.4	119.8	-38.0	-38.7
$frag_1$-$frag_3$	-123.8	-24.2	138.9	-40.0	-49.0
$frag_2$-$frag_3$	-53.8	-19.5	108.1	-38.3	-3.5

Table S4: Interaction energy decomposition analysis (kcal/mol) between the six peptides constituting the hexamer assembly, prototype of amyloid peptide (see fig. 4 d,e,f)

Fragments	E_{ele}	E_{ex}	$\Delta E^{el-prep}$	E_{D3}^{disp}	E_{int}
$frag_1$-$frag_2$	-45.0	-11.1	54.7	-30.0	-31.3
$frag_1$-$frag_3$	-55.0	-12.6	62.2	-32.3	-37.7
$frag_1$-$frag_4$	11.0	-3.1	18.2	-12.7	13.4
$frag_1$-$frag_5$	17.9	0.0	6.5	-0.6	23.8
$frag_1$-$frag_6$	59.6	-0.8	10.9	-1.7	68.0
$frag_2$-$frag_3$	-63.8	-13.8	70.9	-31.0	-37.6
$frag_2$-$frag_4$	33.9	0.0	3.7	-0.3	37.2
$frag_2$-$frag_5$	12.2	-2.1	14.4	-10.8	13.6
$frag_2$-$frag_6$	14.9	0.0	5.8	-0.5	20.2
$frag_3$-$frag_4$	15.5	0.0	5.1	-0.5	20.2
$frag_3$-$frag_5$	43.4	-0.9	10.8	-2.0	51.3
$frag_3$-$frag_6$	10.2	-3.2	19.9	-12.3	14.6
$frag_4$-$frag_5$	-48.0	-13.4	65.4	-30.4	-26.4
$frag_4$-$frag_6$	-56.9	-11.2	55.2	-30.8	-43.6
$frag_5$-$frag_6$	-54.4	-12.5	63.4	-31.3	-34.8
